# Identifying a new “nitrate master”: ZmEREB97 regulates nitrate uptake in maize

**DOI:** 10.1093/plphys/kiae296

**Published:** 2024-05-22

**Authors:** Munkhtsetseg Tsednee

**Affiliations:** Assistant Features Editor, Plant Physiology, American Society of Plant Biologists; Agricultural Biotechnology Research Center, Academia Sinica, Taipei 11529, Taiwan

Nitrogen (N) is an essential nutrient for plants but poses serious challenges in its use for agriculture. In high-N-requiring crops, for example, maize (*Zea mays* L.), N limitation lowers grain yields (Swarbreck et al. 2019). Unfortunately, applying excess N fertilizer cannot always be the solution because it is a source of pollution in the form of the greenhouse gas nitrous oxide and by excess N leaching into soil and water (Schulte-Uebbing et al. 2022).

Cultivated globally for food, biofuel sources, and feedstock, maize accounts for one of the most important crops today. However, like most cereal crops, maize takes up only 30% to 60% of the N fertilizer provided (Zhang et al. 2015). Therefore, there is an urgent need to understand maize N responses in order to improve its N use efficiency (NUE).

In this issue of *Plant Physiology*, Wu et al. (2024) investigated the N responses in 2 maize lines and identified a transcription factor (TF) involved in the regulation of nitrate uptake. First, the authors looked at gene expression in response to nitrogen recovery after depletion. By correlating gene coexpression network data with nitrate-supplied time points, they successfully identified 4 consensus N-related modules conserved in 2 representative maize lines: B73 and Mo17.

Next, they conducted promoter sequence analysis for de novo motif predictions and obtained 6 motifs unique to these 4 modules. One of these highly enriched motifs contains a GCC-box sequence, which is a binding site of AP2/ERF (APETALA2/ETHYLENE RESPONSE FACTOR) family TFs. Using yeast 1-hybrid screening and an electrophoretic mobility shift assay, the authors confirmed that one of the candidate TFs, ZmEREB97, interacts with the selected motif.

Furthermore, *ZmEREB97* mRNA and protein accumulation in roots increases rapidly, in 5 minutes, upon nitrate supply. These studies suggest that ZmEREB97 has a potential role in nitrate response, leading the authors to explore its downstream targets and functions.

By employing DNA affinity purification sequencing, the authors were able to identify 1446 genes, including 130 genes in the previously identified N-response modules, as the targets of ZmEREB97. Additionally, RNA sequencing results obtained from *zmereb97* mutants showed that of the potential target genes, more than one-half were differentially regulated in the mutants than the wild type, supporting the function of ZmEREB97 as an important regulator in N response and metabolism.

Moreover, *zmereb97* mutants accumulate biomass more slowly than wild-type plants both under nitrate-limited and fully nitrate-supplied conditions, and mutants produce fewer grains, with 13% to 15% reductions in grain yields compared with wild-type plants under soil growth conditions (Fig. A and B). As expected, *zmereb97* mutant roots accumulate less nitrate and total N contents due to the lowered net nitrate uptake and influx rate into their roots measured by the flux kinetics analysis. These studies confirm that functional ZmEREB97 is required for nitrate uptake and accumulation and for maintaining growth and yield.

**Figure.**
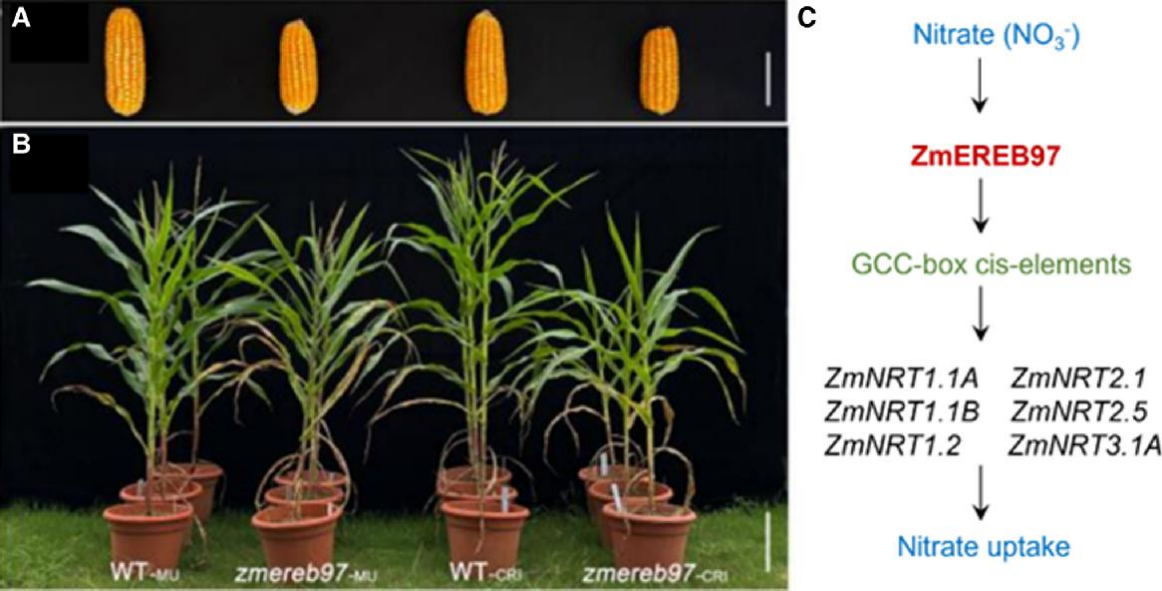
Growth of *zmereb97* mutants and a conceptual diagram of ZmEREB97 regulating nitrate uptake. **A)** and **B)** Phenotype of ear and aboveground tissues of wild-type (WT-_MU_ and WT-_CRI_) and *zmereb97* mutants (*zmereb97-*_MU_ and *zmereb97-*_CRI_ for Mu insertional and CRISPR/Cas9 edited mutants, respectively) in soil conditions. Scale bars = 6 cm in A and 35 cm in B [adapted from Wu et al. (2024), Figure 6K and 6L]. **C)** Conceptual diagram of ZmEREB97 showing its positive regulation of nitrate transporter genes *ZmNRT1.1A*, *ZmNRT1.1B*, *ZmNRT1.2*, *ZmNRT2.1*, *ZmNRT2.5*, and *ZmNRT3.1A* via direct binding to GCC-box cis-elements in their promoters to enhance nitrate uptake. Arrows indicate positive connections to the process.

To reveal how ZmEREB97 regulates nitrate uptake, the authors conducted yeast 1-hybrid assays using 17 selected nitrate transporter (*NRT*) genes potentially regulated by ZmEREB97 and showed that 6 *ZmNRT*s interact with ZmEREB97 via GCC-elements in their promoters for transcriptional activation. These 6 are the transporters mainly responsible for nitrate uptake from soil (Fig. C). Direct controlling of uptake transporters is vital because it is the primary source of nutrients into roots. Therefore, Wu et al. (2024) have identified a critical player, ZmEREB97, as a major positive regulator in nitrate response in maize.

These findings extend our understanding of the AF2/ERF family TFs for their crucial roles in regulating nitrate uptake and utilization in plants. Previously, members of the AP2/ERF TF family were shown to upregulate *NRT* gene expressions in Arabidopsis (Zhang et al. 2014) and rice (Wei et al. 2022). Furthermore, this study identified a promising target, *ZmEREB97*, for further efforts to improve the NUE in maize.
